# Physical Examination Tools Used to Identify Swollen and Tender Lower Limb Joints in Juvenile Idiopathic Arthritis: A Scoping Review

**DOI:** 10.1155/2018/3408162

**Published:** 2018-05-15

**Authors:** Antoni Fellas, Davinder Singh-Grewal, Derek Santos, Andrea Coda

**Affiliations:** ^1^School of Health Sciences, Faculty of Health and Medicine, University of Newcastle, Callaghan, NSW, Australia; ^2^The Sydney Children's Hospital, Randwick, Sydney, NSW, Australia; ^3^The Children's Hospital at Westmead, Sydney, NSW, Australia; ^4^Discipline of Paediatrics and Child Health, University of Sydney, Sydney, NSW, Australia; ^5^Discipline of Paediatrics, Western Sydney University, Sydney, NSW, Australia; ^6^School of Health Sciences, Queen Margaret University, Edinburgh EH21 6UU, UK

## Abstract

**Background:**

Juvenile idiopathic arthritis (JIA) is the most common form of rheumatic disease in childhood and adolescents, affecting between 16 and 150 per 100,000 young persons below the age of 16. The lower limb is commonly affected in JIA, with joint swelling and tenderness often observed as a result of active synovitis.

**Objective:**

The objective of this scoping review is to identify the existence of physical examination (PE) tools to identify and record swollen and tender lower limb joints in children with JIA.

**Methods:**

Two reviewers individually screened the eligibility of titles and abstracts retrieved from the following online databases: MEDLINE, EMBASE, Cochrane Central Register of Controlled Trials, and CINAHL. Studies that proposed and validated a comprehensive lower limb PE tool were included in this scoping review.

**Results:**

After removal of duplicates, 1232 citations were retrieved, in which twelve were identified as potentially eligible. No studies met the set criteria for inclusion.

**Conclusion:**

Further research is needed in developing and validating specific PE tools for clinicians such as podiatrists and other allied health professionals involved in the management of pathological lower limb joints in children diagnosed with JIA. These lower limb PE tools may be useful in conjunction with existing disease activity scores to optimise screening of the lower extremity and monitoring the efficacy of targeted interventions.

## 1. Background

Juvenile idiopathic arthritis (JIA) affects between 16 and 150 per 100,000 young persons below the age of 16 [1], with the lower limb being commonly involved [2–5]. The hip, knee, and ankle are the most commonly affected lower limb joints in JIA, with prevalence rates of 30–50% across all subtypes [3, 6, 7]. Approximately 40% of JIA patients will experience rear foot (i.e., subtalar) joint synovitis [8], while the midfoot (talonavicular and calcaneocuboid) and phalangeal joints appear to be less affected [5]. The International League of Associations for Rheumatology guideline for the diagnosis of JIA relies on clinical examination and the number of joints affected, forming part of the classification criteria [9]. Early diagnosis and treatment are the gold-standard approach in paediatric rheumatology [10]. However, evidence suggests that there are significant delays in diagnosis [11–13], with a recent study conducted in Australia reporting that more than 40% of children with JIA had a delay of 5 or more months between onset of symptoms and diagnosis [13]. This delay in diagnosis is likely to have a negative impact on the long-term health outcomes in children with JIA [14].

Lower limb joint involvement in JIA consists of active arthritis and joint damage. Swelling of foot and ankle joints, particularly the rear foot and midfoot joints, may not be clinically evident at disease onset and may present at a later stage as the disease flares or worsens. Moreover, systemic pharmaceuticals and intracorticosteroid injections are effective interventions in JIA but may not cause complete remission of active arthritis in joints [1]. Prolonged active arthritis may increase the risk of permanent joint damage, such as cartilage erosion [15]. This may lead to increased pain, impaired joint function, and higher rates of orthopaedic intervention, such as joint arthroplasty [15, 16]. Two long-term outcome studies in JIA, which include 328 participants in total, have shown that the lower limb required more surgical interventions than the upper limb [15, 17]. The most commonly operated lower limb joints were the hip, knee, and ankle [15, 17]. Identifying lower limb active arthritis earlier and frequent screening may reduce the risk of irreversible joint damage and the need for surgical intervention.

Recent evidence highlighted the potential limitations of physical examination (PE) in JIA, with studies reporting that medical imaging such as ultrasound and magnetic resonance imaging may be more accurate in detecting active arthritis, particularly subclinical disease [3, 7, 18–20]. Despite this limitation, careful and routine PE remains gold standard and an important assessment to detect early clinical changes and prompt localised interventions. Paediatric rheumatologists conduct both upper and lower limb PE of joints as part of their routine clinical assessment. Allied Health Professionals (AHPs) such as podiatrists who focus solely on the lower extremity may assist paediatric rheumatologists in providing an additional screening of lower limb joints using a standardised lower limb PE tool. This may allow for a standardised and systematic method of screening the lower extremity for early detection of active joint disease. It may also enhance the ability of the paediatric rheumatology team to detect active arthritis in more difficult to assess joints such as the rear foot and midfoot joints of the feet. Lower limb tools may resemble a manikin or tabular form, depicting a focused count of swollen and tender lower limb joints. Moreover, AHPs may also be involved in providing localised, safe, noninvasive physical and mechanical therapies that may implement patients' current medical management. For example, foot orthoses may be prescribed to reduce lower limb pain and improve quality of life [21, 22]. Finally, a PE tool specific to the lower limb may enable clinicians and researchers in allied health to test the effectiveness of localised interventions in reducing swelling and tenderness of lower extremity joints.

Scoping reviews may be used to search the literature to thoroughly identify research gaps, provide summaries, and justify the feasibility of conducting a future systematic review [23]. This scoping review aims to identify the existence of lower limb PE tools in detecting swollen and tender lower limb joints in JIA.

## 2. Objective

The objective of this scoping review is to identify the existence for PE tools that may be used to detect and record swollen and tender lower limb joints in children with JIA.

## 3. Methods

According to Arksey and O'Malley (2005), a scoping review is a comprehensive search of the evidence, which aims at identifying existing gaps in the scientific literature [23]. A scoping review can determine the value of undertaking a systematic review, whether it is feasible or not based on current available literature, or if a systematic review has already been conducted [23, 24]. Lastly, despite methodological differences between scoping and systematic reviews, scoping reviews may summarise and disseminate key research findings [25]. For this paper, a scoping review was undertaken to explore the literature on the existence of PE tools for the detection of swollen and tender lower limb joints in children diagnosed with JIA. A recently updated methodological framework for scoping reviews published by Khalil et al. (2016) was adopted [25].

### 3.1. Types of Studies Included

- Studies that involved designing or introducing a PE protocol or tool for the identification of lower limb joint swelling and tenderness in JIA were included in this study.
- Studies that involve disability tools were excluded. Disability tools will measure the physical impact that active arthritis has on lower limb joints. The purpose of this scoping review is to identify standardised PE tools that assist in the detection of active arthritis rather than tools that measure physical disability.

### 3.2. Types of Physical Examination Tools

- PE tools that aid in identifying and recording swollen and tender lower limb joints affected by active disease in JIA were included.
- Lower limb joints included in PE tools may contain the hip, knee, ankle, subtalar (rear foot), talonavicular and calcaneocuboid (midfoot), metatarsophalangeal (forefoot), and proximal and distal interphalangeal joints (digits).
- Any design of the tool itself, which may contain a manikin or tabular form, was also included.
- Tools that consisted of an upper limb joint count were excluded.

#### 3.2.1. Searches

The MEDLINE (January 1966 to August 2016) search strategy is presented in Table 1. This search strategy was adapted for EMBASE (January 1980 to August 2016), Cochrane Central Register of Controlled Trials (CENTRAL) (the Cochrane Library, latest issue), and CINAHL (from 1982). No language or publication restrictions were applied. Reference lists of all included studies were checked for other potentially eligible papers.

Two reviewers (Antoni Fellas and Andrea Coda) individually screened the titles and abstracts of all studies identified by the search strategy. Full-text papers of potentially eligible studies were retrieved by Antoni Fellas and individually screened by Antoni Fellas and Andrea Coda. If the two reviewers (Antoni Fellas and Andrea Coda) did not resolve disputes successfully, a third reviewer (Derek Santos) would act as an arbitrator to resolve any disagreements, though this was never required.

#### 3.2.2. Data Extraction/Charting

As no studies were included in this scoping review, a full data extraction was not conducted. Extraction of information from included studies was to be as follows: author(s), year of publication, country of origin, aims, study population, sample size, methodology, type of PE tool, type of comparator (if applicable), how outcomes are measured, and key findings. To assist in the interpretation of results, basic descriptive information of potentially eligible studies was obtained and presented in Table 2. Charting the data in this paper included a combination of narrative style writing and the presentation of a summary table.

## 4. Results

### 4.1. Description of Studies

#### 4.1.1. Studies Identified

**Figure 1:**
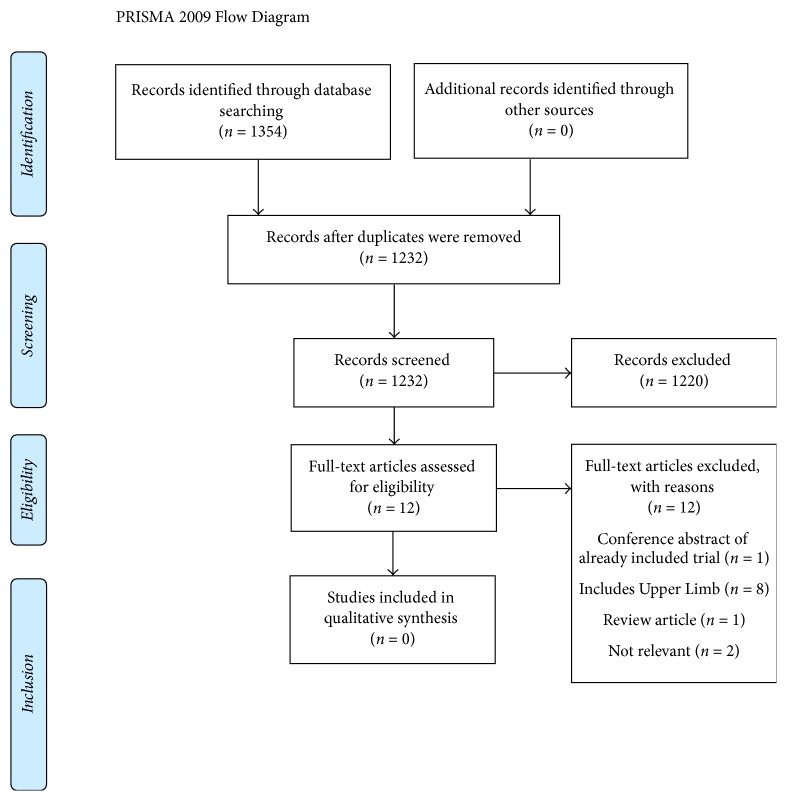
PRISMA flow diagram.

After the removal of duplicates, 1232 studies were retrieved from the search (Figure 1). Twelve potentially eligible papers were identified after titles and abstracts were screened, for which full texts were retrieved [26–37]. One paper was immediately excluded as it was a conference abstract of an already included paper [37]. Seven papers were excluded as they involved an upper limb joint count in either an overall global disease activity score or PE tool [27–29, 31, 32, 35, 36]. One study was excluded, as it was a review article [30]. Antoni Fellas screened all references for additional suitable PE tools; however, no additional papers were retrieved. Two potentially eligible studies compared clinical examination to ultrasound in the identification of pathological joints in JIA [33, 35]. The objective of these studies was not to develop or validate a lower limb PE tool; therefore, they were excluded. One more study was excluded as it was not relevant [34]. Basic data on the potentially eligible papers are outlined in Table 2.

### 4.2. Juvenile Arthritis Foot Disability Index

Only one study identified as potentially eligible was specific to the lower limb [26]. The authors of the study developed a foot and ankle disability index for JIA, called the juvenile arthritis foot disability index (JAFI). The JAFI measures foot and ankle disability using 27 consecutive questions regarding the effects of arthritis on their physical impairment, activity limitation, and participation restriction. The authors concluded that the JAFI is a valid and reliable measure of foot and ankle disability in children and adolescents with JIA [26]. Overall, the design and the purpose of the study were not to validate a PE tool for lower limb joint swelling and tenderness in JIA. Thus, the study does not fit the inclusion criteria for this scoping review and was ultimately excluded.

### 4.3. Summary of Existing Tools

This section will summarise how the lower limb was assessed in those studies listed as potentially eligible and relevant.

- Two studies used a 69-joint, full body manikin including joints from both upper and lower limbs [27, 32]. The 69-joint count included the hip, knee, ankle, metatarsophalangeal joints, and phalanges of the feet but not the subtalar and midfoot joints and did not distinguish between distal and proximal phalangeal joints. The 69-joint manikin was designed to test joint examination by patients and parents of active disease in JIA and compare their assessments to paediatric rheumatologists' assessments [27]. This may account for why more difficult clinical examinations (such as the rear foot and midfoot joints) were excluded.
- A 67-joint count was used by one study to develop weighted scores in JIA [28]. The type of PE tool was unclear and the study did not include the subtalar and midfoot joints and did not distinguish between distal and proximal phalangeal joints [28].
- One study aimed to develop and test reduced joint counts. Four different reduced joint counts were tested, in which all of them did not include the subtalar, midfoot, and interphalangeal joints [29]. It was also unclear what type of PE tool was used.
- One study aimed to develop and validate joint damage in JIA [36]. Part of the index included clinical range of motion testing for the hip, knee, ankle, and metatarsophalangeal joints, as well as scores for fixed flexion deformities that may or may not have required surgical intervention [36]. The subtalar, midfoot, and phalangeal joints were not included in this joint damage index.
- Lastly, one study aimed to develop and validate a composite score for JIA called the Juvenile Arthritis Disease Activity Score (JADAS) [31]. Part of this composite score included simple joint counts for active disease of varying number. The 71-joint count included the largest number of joints covered in both upper and lower extremities. The JADAS-71 includes all lower limb joints except the distal and proximal phalangeal joints, and it is unclear what midfoot joints were included [31].

Overall, these studies did not include the rear foot and midfoot joints, as well as distal and proximal phalangeal joints in their joint assessments or PE tools. The outcome of this finding needs to be taken into context with the aims of the studies outlined in this section. For example, majority of these studies focused on developing and validating reduced joint counts or disease composite scores; thus the exclusion of certain lower limb joints, particularly in the feet, was prudent. The authors of one study concluded that, despite the exclusion of joints such as the subtalar and midfoot joints, it does not deter the importance of screening them for active disease [28].

## 5. Discussion

The results of this scoping review have indicated paucity in the current literature for a validated PE tool in the assessment of lower limb joint swelling and tenderness. Seven potentially eligible papers were excluded from this review as they involved an upper limb count of swollen and tender joints [27–29, 31, 32, 36]. Consolaro et al. (2009) developed the composite disease activity score in JIA, the JADAS [31]. This extensively validated tool is used in children with JIA, representing the most widely recognised and accepted global activity scale in paediatric rheumatology. The JADAS is commonly used to record global disease changes, which includes varying counts of upper and lower swollen and tender (active) joints [31, 38]. Certain physical and mechanical therapies such as foot orthoses target specific areas of the body (i.e., the lower limb only) and they are not prescribed as a stand-alone intervention to impact on overall disease activity. Therefore, the JADAS may not be suitable in effectively measuring the impact that therapies such as foot orthoses have on the lower limb joint swelling and tenderness. Helliwell (2007) suggested a PE tool for swollen and tender foot and ankle joints for adult rheumatoid arthritis [39]. The proposed PE tool is a 14-joint count (28 bilaterally), which includes the ankle, subtalar, talonavicular, calcaneocuboid, metatarsophalangeal, and interphalangeal joints [39]. A modified version of this tool, which also includes the hip, knee, and distal and proximal interphalangeal joints, may be appropriate for establishing the reliability and validity in future studies.

This scoping review focused on PE as a diagnostic tool to identify swollen and tender lower limb joints in JIA. Upper limb joint counts were excluded in this review as the objective of our research was to identify validated lower limb PE tools, which may be used for future clinical research, focusing on physical and/or mechanical therapies for lower extremity problems in JIA. Moreover, a review to identify the existence of upper limb PE tools may be of benefit for those focusing on therapies targeting upper limb problems only in JIA. A limitation of this scoping review is that joint damage and musculoskeletal problems secondary to active arthritis were not considered in this study but, nevertheless, are important clinical problems to screen in JIA. The paediatric gait, arms, legs, and spine (pGALS) is a validated screening tool in paediatrics and can be used by paediatric rheumatologists to identify joint and musculoskeletal abnormalities in children with JIA [40]. Finally, this scoping review revealed that currently there are no eligible studies according to our inclusion criteria. These findings justify even further that a systematic review may not have been feasible for this specific research question. Further research in designing and validating optimal lower limb PE tools in JIA is needed.

Future studies may focus on testing validity and reliability (intra- and interrater) of a lower limb PE tool compared to a sensitive imaging modality such as magnetic resonance imaging. These easily accessible tools can be utilised by AHPs, as part of the daily clinical assessment, accompanied by additional validated measures, such as the pGALS or JADAS. This may promote the early detection of both active disease and musculoskeletal problems in the lower extremity with children suffering from JIA. Lastly, validated and reliable PE tools specific to the lower limb may be useful for researchers investigating the efficacy of therapies for the lower extremity in JIA.

## 6. Conclusion

This scoping review did not identify any validated PE tools for the count of swollen and tender lower limb joints in JIA. Further research may aim to develop and validate future lower limb PE tools, which may be used in combination with other validated measures of disease activity in a multidisciplinary approach to detect lower limb joint disease. Moreover, clinicians specialising in the lower limb such as podiatrists and other AHPs may find a validated tool useful to monitor the progress of targeted interventions.

## Figures and Tables

**Table 1:** OvidSP MEDLINE search strategy.

(1) Juvenile idiopathic arthritis.mp. or Arthritis, Juvenile/
(2) Juvenile rheumatoid arthritis.mp.
(3) Juvenile chronic arthritis.mp.
(4) (1) or (2) or (3)
(5) exp Physical Examination/
(6) (joint^*∗*^ adj3 (exam^*∗*^ or assess^*∗*^)).mp.
(7) (5) or (6)
(8) (4) and (7)

**Table 2:** Summary of potential eligible studies.

Author(s)	Year	Aim	Sample size	Type of PE tool	Joint count	Included (yes/no)
Andre et al.	2004	To develop a new juvenile arthritis foot disability index (JAFI) and to test it for validity and reliability	36 JIA participants; 29 controls	Unclear	Foot and ankle disability questionnaire	No

Anink et al.	2014	Conference abstract paper of Dijkstra et al. (2015)	—	—	-	No

Armbrust et al.	2013	To investigate the assessment by patients and parents of disease activity in JIA and compare against rheumatologist	113 JIA participants	Full body manikin, PE tool. Includes both lower and upper limbs	69-joint count Lower and upper limbs	No

Bandeira et al.	2006	Developing a set of weighted scores for joint counts in JIA	121 JIA participants	Unclear	67-joint count Lower and upper limbs	No

Bazso et al.	2009	To develop and test reduced joint counts in children with JIA	Retrospective design: total of 4353 participants	Simple joint count unclear on type of tool (i.e., manikin/figure/form)	Four different joint counts: 45, 35, 27, and 10 Lower and upper limbs	No

Consolaro et al.	2009	To develop and validate a composite disease activity score for JIA, the Juvenile Arthritis Disease Activity Score (JADAS)	Retrospective design: total of 4578 participants	Simple joint count unclear on type of tool (i.e., manikin/figure)	Three different joint counts: 71, 27, and 10 Lower and upper limbs	No

Consolaro et al.	2014	Review article	—	—	—	No

Dijkstra et al.	2015	To evaluate reliability of a manikin-format patient reported joint count in JIA	75 JIA participants	Full body manikin, PE tool. Includes both lower/upper limbs	69-joint count (same PE tool as Dijkstra) Lower and upper limbs	No

Hendry et al.	2012	Agreement of clinical examination versus ultrasonography of foot disease in JIA	30 JIA participants	Unclear	24-joint count (12 each side) Foot and ankle only	No

Magni-Manzoni et al.	2005	Not relevant	—	—	-	No

Magni-Manzoni et al.	2009	To compare clinical evaluation and ultrasonography in the assessment of joint synovitis in JIA	32 JIA participants	Unclear	52-joint count Lower and upper limbs	No

Viola et al.	2005	To develop and validate a clinical measure of articular and extra-articular damage in patients with JIA	158 JIA participants	Simple joint count unclear on type of tool (i.e., manikin/figure)	67-joint count Lower and upper limbs	No

## Data Availability

Data sharing is not applicable to this article as no datasets were generated or analysed during the current study.

## Abbreviations

JIA: Juvenile idiopathic arthritis
PE: Physical examination
AHPs: Allied Health Professionals
JAFI: Juvenile arthritis foot disability index
JADAS: Juvenile Arthritis Disease Activity Score
pGALS: Paediatric gait, arms, legs, and spine.
